# Physicochemical Characterization and Antioxidant and Hypolipidaemic Activities of a Polysaccharide From the Fruit of *Kadsura coccinea* (Lem.) A. C. Smith

**DOI:** 10.3389/fnut.2022.903218

**Published:** 2022-05-18

**Authors:** Hairong Long, Xianghua Xia, Suqi Liao, Tao Wu, Lijun Wang, Qianping Chen, Shugen Wei, Xiaoyu Gu, Zhenjun Zhu

**Affiliations:** ^1^Guangxi Botanical Garden of Medicinal Plants, No. 189, Nanning, China; ^2^Key Laboratory of Food Nutrition and Safety, Ministry of Education, Tianjin Key Laboratory of Food Nutrition and Safety, Tianjin University of Science and Technology, Tianjin, China; ^3^Institute of High Energy Physics, Chinese Academy of Sciences, Beijing, China; ^4^Department of Food Science and Engineering, College of Science and Engineering, Jinan University, Guangzhou, China

**Keywords:** structural characterization, antioxidant activity, *Kadsura coccinea* fruit, polysaccharide, hypolipidaemic activity

## Abstract

*Kadsura coccinea* fruit, a novel fruit resource, has attracted wide interest, but the physicochemical characteristics and biological activities of its polysaccharides remain unclear. This study investigated the physicochemical properties of a polysaccharide extracted from *K. coccinea* fruit polysaccharide (KCFP) and evaluated its antioxidant and hypolipidaemic activities *in vitro* and *in vivo*. KCFP is an amorphous, thermally stable pectin heteropolysaccharide with an average molecular weight of 204.6 kDa that is mainly composed of mannose, rhamnose, glucose, galactose, xylose, arabinose, galacturonic acid (molar percentage >70%) and glucuronic acid. 2,2-Diphenyl-1-picrylhydrazyl (DPPH) and 2,2′-azino-bis(3-ethylbenzothiazoline-6-sulfonic acid) (ABTS) free radical scavenging assays and an iron reducing antioxidant power assay showed that KCFP has strong antioxidant capacity, while the bile acid binding assay showed that KCFP has hypolipidaemic potential *in vitro*. The antioxidant and hypolipidaemic activities of KCFP were further evaluated in high-fat diet-induced hyperlipidaemic mice. KCFP significantly increased the activities of superoxide dismutase, glutathione peroxidase and catalase, decreased the malondialdehyde content, significantly reduced the total cholesterol (TC), triglyceride (TG) and low-density lipoprotein cholesterol (LDL-C) levels, and increased the amount of high-density lipoprotein cholesterol (HDL-C). These findings suggest that KCFP could be used as a functional food to remedy oxidative damage and hyperlipidaemia.

## Introduction

Hyperlipidaemia is caused by abnormal lipid metabolism or transport, which leads to increases in lipid levels in plasma and multiple tissues, is a major factor contributing to fatty liver disease, hyperlipidaemia and diabetes (1). Dyslipidaemia is an important factor influencing cardiovascular disease that has become a global health problem (2). In clinical practice, synthetic compounds used to treat hyperlipidaemia, including statins and fibrates, cause many side effects (3). Therefore, research and development on natural medicines for the prevention and treatment of hyperlipidaemia and its complications are urgently needed.

Polysaccharides are important natural macromolecules with unique physicochemical properties and biological properties, such as immunoregulatory, antitumour, antioxidant, hypoglycaemic, antiglycation, and anti-fatigue activities. In recent years, plant-derived polysaccharides have received extensive attention from researchers (4).

*Kadsura coccinea* (Lem.) A. C. Smith is a vine that is mainly distributed in southern and southwestern China, Vietnam and Myanmar. The roots and stems of *K. coccinea* are traditional medicines that are commonly and widely used among the Zhuang and Yao populations in China. The peculiar, aggregate fruits of *K. coccinea* consist of 30 to 70 separate carpels. Once ripe, the fruits are red or dark purple and typically 6 to 10 cm in diameter. As nonmedicinal parts of the plant, *K. coccinea* fruits have not been well developed or utilized and are even discarded. Our previous studies showed that *K. coccinea* fruits contain a variety of nutrients, including important polysaccharides (5). To date, there have been no published reports on the physicochemical properties and biological activities of a specific *K. coccinea* fruit polysaccharide (KCFP).

Hence, the purpose of this study was to elucidate the physicochemical properties of water-extracted KCFP and investigate its antioxidant and hypolipidaemic activities *in vitro* and *in vivo*. This research will provide a reference for the scientific utilization of *K. coccinea* plant resources.

## Materials and Methods

### Materials and Chemicals

*Kadsura coccinea* fruit was collected from a research farm in Guangxi, China. Monosaccharide standards and dextrans with different molecular weights were purchased from the National Institute for Food and Drug Control (Beijing, China). 2,2-Diphenyl-1-picrylhydrazyl (DPPH), 2,2′-azino-bis(3-ethylbenzothiazoline-6-sulfonic acid) diammonium salt (ABTS), cholic acid (CA), deoxycholic acid (DCA), taurocholic acid (TCA) and glycocholic acid (GCA) were purchased from Macklin (Shanghai, China). 1-Phenyl-3-methyl-5-pyrazolone (PMP), pancreatin and cholestyramine resin were purchased from Sigma–Aldrich (St. Louis, MO, United States). High-fat chow and regular chow were purchased from Beijing Keaoxieli Feedstuff Co., Ltd. (Beijing, China). Assay kits for total cholesterol (TC), triglyceride (TG), low-density lipoprotein cholesterol (LDL-C), high-density lipoprotein cholesterol (HDL-C), glutathione peroxidase (GSH-Px), malondialdehyde (MDA), total superoxide dismutase (SOD) and catalase (CAT) were obtained from the Nanjing Jiancheng Bioengineering Institute (Nanjing, Jiangsu, China). All other reagents used in the present study were of analytical grade.

### Extraction and Purification of *Kadsura coccinea* Fruit Polysaccharide

The fruits were dried at 50°C with hot air, crushed into a powder, and passed through a 40-mesh sieve. One hundred grams of dried fruit powder was degreased and decolorized with 95% ethanol. The filtered residue was dissolved in distilled water (1 L) and stirred at 95 °C under reflux for 4 h. The reaction mixture was centrifuged (8000 *g*, 15 min) twice, and the combined supernatants were concentrated to 200 mL with a rotary evaporator. The solution was precipitated with a final concentration of 80% ethanol, and the precipitate was washed sequentially with absolute ethanol, acetone, and anhydrous ether and then dissolved in water to remove free protein using the Sevag method. The sample was then dialyzed (Mw cut-off 3500 Da) and lyophilized to obtain KCFP (4.2 g). The obtained KCFP was analyzed using high-performance liquid chromatography with an evaporative light scattering detector (HPLC-ELSD; Agilent 380ELSD, United States) in combination with a TSKgel-G5000 PW_XL_ gel chromatography column to confirm homogeneity.

### Analysis of the Physical and Chemical Properties of *Kadsura coccinea* Fruit Polysaccharide

#### Analysis of the Chemical Composition

The total carbohydrate content of KCFP was measured according to the phenol−sulfuric acid method. The total protein content was measured according to the Bradford method. The uronic acid content was determined using the sulfuric acid-carbazole method, using galacturonic acid (GalA) as the standard. The sulfate content was determined with ion chromatography (DIONEX ICS-90, United States). The contents of C, H, and N were detected with a CHN628 elemental analyser (LECO, United States), and the S content was determined with a Multi EA4000 elemental analyser (Jena, Germany).

#### Determination of the Average Molecular Weight

The average molecular weight of KCFP was estimated using gel permeation chromatography (GPC) (Waters-Alliance, United States) with a TSK G5000 PW_XL_ column, an e2695 separation module, and a 2414 refractive index detector. The sample injection volume was 50 μL (2 mg/mL), distilled water (pH 7.0) was used as the mobile phase, the flow rate was 0.6 mL/min, and the column temperature was 60°C. The molecular weight was estimated by referring to a calibration curve prepared from a standard dextran series (Mw values of 4.32, 12.6, 73.8, 126, 289, and 496 kDa).

#### Analysis of the Monosaccharide Composition

The monosaccharide composition was measured using HPLC (Agilent 1260, United States) with PMP precolumn derivatization as described in a previous study (6).

#### Thermogravimetry and X-Ray Diffraction Analysis

The thermal properties of KCFP were determined by thermogravimetry and derivative thermogravimetry, and thermal analysis curves were obtained by simultaneous thermal analysis with an STA449F5 instrument (NETZSCH, Germany). KCFP (5 mg) was transferred to an alumina crucible for thermogravimetry analysis under a nitrogen atmosphere at a heating rate of 10 °C/min in the range of 30–800°C.

X-ray diffraction (XRD) patterns were obtained on a D/max-2500 diffractometer (Rigaku, Japan). The diffractometer had a scanning angle of 5–90° and a scanning rate of 5°/min.

#### Morphological Observations

The lyophilized polysaccharide powder was affixed to the sample stage using double-sided conductive tape, and images were captured at different magnifications using a Phenom Pro scanning electron microscope (SEM, Phenom-World, Netherlands). The KCFP sample was completely dissolved in pure water at a concentration of 5 μg/mL, and 5 μL of the solution was dropped on a freshly cleaved mica surface to acquire atomic force microscopy (AFM) images. After air drying, the morphology was observed using a Dimension Edge AFM (Bruker, Germany).

#### Fourier Transform Infrared Spectral Analysis

*Kadsura coccinea* fruit polysaccharide was mixed with dry KBr (1:50) and pressed into transparent films. The films were scanned using a Tensor II fourier transform infrared (FTIR) spectrometer (Bruker, Germany) from 4000 to 400 cm^–1^.

#### Nuclear Magnetic Resonance Analysis

^1^H and ^13^C nuclear magnetic resonance (NMR) spectra of KCFP were acquired using a 600 MHz NMR spectrometer (Varian, United States). Briefly, after drying the KCFP at 50 °C to constant weight, approximately 15 mg of sample was dissolved in 0.6 mL of 99.98% D_2_O, and the ^1^H NMR spectrum was collected using the PRESAT water signal suppression method described by the manufacturer. In addition, approximately 30 mg of KCFP was dissolved in 0.6 mL of 99.98% D_2_O for ^13^C NMR spectroscopy. Each sample was scanned 1024 times for ^1^H NMR analysis and 70000 times for ^13^C NMR analysis.

#### Rheological Properties

The rheological properties of a 5 mg/mL KCFP aqueous solution were determined at 25 ± 0.1°C with a HAAKE MARS40 rheometer (Thermo, Germany) equipped with a P35 parallel plate (35 mm in diameter, gap of 0.5 mm). For each measurement, 1 mL of sample solution was carefully loaded onto the Peltier plate of the rheometer. The flow curve was obtained at 25 °C with shear rates ranging from 0.1 to 1000 s^–1^. A frequency sweep from 0.1 to 10 Hz was conducted at 25°C at a constant stress within the region of linear viscoelasticity.

### Antioxidant and Hypolipidaemic Activities of *Kadsura coccinea* Fruit Polysaccharide *in vitro*

#### 2,2-Diphenyl-1-Picrylhydrazyl Radical Scavenging Activity

The ability of KCFP to scavenge DPPH free radicals was analyzed as previously described (7). Briefly, 0.1 mL aliquots of sample solutions at different concentrations (0.05, 0.1, 0.25, 0.5, and 1.0 mg/mL) were transferred to a 96-well plate. Then, 0.1 mL of DPPH in ethanol (0.1 mmol/L) was added to each well of the plate, and the mixtures were stirred well. The plate was incubated at room temperature for 30 min in the dark. Trolox served as the positive control. The absorbance was measured using an Infinite 200 PRO microplate reader (Tecan, Switzerland) at 517 nm. The DPPH radical scavenging activity was calculated using the following formula:


(1) $$\mathrm{Scavenging}\mathrm{rate}\left(\%\right)=\left(1-\frac{A_{2}-A_{1}}{A_{0}}\right)\times 100$$

where, A_1_ is the absorbance of the sample solution after reaction with solvent (distilled water); A_2_ is the absorbance of the sample solution after reaction with DPPH; and A_0_ is the absorbance of the solution containing solvent (distilled water) and DPPH.

#### ABTS Radical Scavenging Assay

The ABTS radical scavenging activity of KCFP was assessed using a reported procedure (8) with some modifications. ABTS (7.4 mmol/L) was mixed with an equal volume of a 2.6 mmol/L K_2_S_2_O_8_ solution to obtain an ABTS^+^ stock solution. The stock solution was stored in the dark for 12–16 h and then diluted with deionized water to obtain a working solution; the absorbance of the working solution at 734 nm was adjusted to 0.70. Then, 0.05 mL of various concentrations of KCFP solution (0.05, 0.1, 0.25, 0.5, and 1.0 mg/mL) were added to 0.2 mL of the ABTS^+^ working solution. After reacting for 6 min at 25°C, the absorbance was measured at 734 nm. Trolox was used as the positive control. The ABTS radical scavenging activity was calculated with the following equation:


(2) $$\mathrm{Scavenging}\mathrm{rate}\left(\%\right)=\left(1-\frac{A_{2}-A_{1}}{A_{0}}\right)\times 100$$

where, A_1_ is the absorbance of the sample solution after reaction with solvent (distilled water); A_2_ is the absorbance of the sample solution after reaction with the ABTS^+^ working solution; and A_0_ is the absorbance of the solution containing solvent (distilled water) and the ABTS^+^ working solution.

#### Ferric Reducing Antioxidant Power

The ferric reducing antioxidant power (FRAP) test was conducted based on published methods (9, 10). A standard curve was prepared using different concentrations (10–500 μmol/L) of FeSO_4_⋅7H_2_O. The antioxidant capacity, based on the ability of the sample to reduce ferric ions, was calculated from the linear calibration curve and is reported as the concentration of sample having a ferric reducing ability equivalent to that of 1 μmol/L FeSO_4_⋅7H_2_O.

#### Bile Acid Binding Capacity

The *in vitro* bile acid binding capacity of KCFP was investigated according to our previously reported method (4). Under simulated gastrointestinal digestion conditions, the ability of KCFP to bind CA, DCA, GCA and TCA was evaluated. The bile acid binding ability of KCFP is expressed in μmol of bile acid per 100 mg of dry matter (DM).

### Antioxidant and Hypolipidaemic Activities of *Kadsura coccinea* Fruit Polysaccharide in High-Fat Diet-Induced Hyperlipidaemic Mice

#### Animal Experimental Design

The *in vivo* antioxidant and hypolipidaemic activities of KCFP were further evaluated in high-fat diet-induced hyperlipidaemic mice. All experimental procedures were performed in accordance with the “Guidelines for the Care and Use of Laboratory Animals: Eighth Edition.” Eighteen male C57BL/6N mice aged 5–6 weeks were purchased from Beijing Vital River Laboratory Animal Technology Co., Ltd. After 1 week of adaptive feeding, the mice were divided into two groups: 6 mice in the normal control group (NC) were fed a basal diet, and 12 mice in the high-fat diet group were fed a high-fat diet. After 8 weeks of feeding, the mice in the high-fat diet group were randomly divided into two groups (*n* = 6), named the model control (MC) group and the KCFP group. Both the MC group and the KCFP group continued to be fed a high-fat diet, and the KCFP group was administered a daily dose of 300 mg KCFP/kg body weight. After 8 weeks of feeding, serum and liver samples from the experimental mice were collected for analysis.

#### Histopathological Examination of Liver Tissues

Liver tissues were cut into 5 μm thick sections and stained with haematoxylin and eosin (H&E). The stained samples were assessed for histopathological changes using a BX43 light microscope (Olympus, Tokyo, Japan).

#### Biochemical Measurements of the Serum and Liver Samples

The relevant antioxidant parameters (SOD, GSH-Px, CAT and MDA) and hypolipidaemic parameters (TC, TG, LDL-C and HDL-C) were measured using assay kits (Nanjing Jiancheng Bioengineering Institute, Nanjing, China).

### Data Processing

The experimental data were processed using SPSS software and are presented as the means ± standard deviation. Data were further analyzed using one-way analysis of variance (ANOVA) and Tukey’s multiple comparison tests. Differences were determined to be significant at *p* < 0.05.

## Results and Discussion

### Analysis of the Physical and Chemical Properties

#### Analysis of the Chemical Composition of *Kadsura coccinea* Fruit Polysaccharide

**FIGURE 1:**
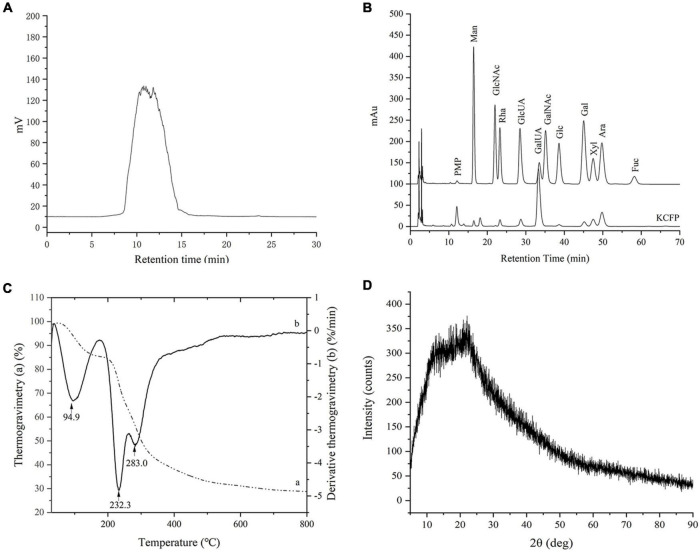
GPC analysis **(A)**, monosaccharide composition **(B)**, thermogravimetry analysis **(C)**, and XRD analysis **(D)** of KCFP. PMP, 1-phenyl-3-methyl-5-pyrazolone; Man, mannose; GlcNAc, N-acetyl-glucosamine; Rha, rhamnose; GlcUA, glucuronic acid; GalUA, galacturonic acid; GalNAc, N-acetyl-galactosamine; Glc, glucose; Gal, galactose; Xyl, xylose; Ara, arabinose; Fuc, fucose; GPC, gel permeation chromatography; XRD, X-ray diffraction; KCFP, *Kadsura coccinea* fruit polysaccharide.

*Kadsura coccinea* fruit polysaccharide was composed of 60.42 ± 1.76% carbohydrates, 2.30 ± 0.26% proteins and 0.48 ± 0.08% sulfate. The elemental N and S contents were analyzed and were found to be consistent with the protein and sulfate contents, respectively. As shown in Figure 1A, the average molecular weight of KCFP was approximately 204.6 kDa. The monosaccharide composition analysis showed that KCFP was a heteropolysaccharide consisting of mainly mannose, rhamnose, glucose, galactose, xylose, arabinose, GalA and glucuronic acid at a molar ratio of 1.00:3.56:1.03:2.01:7.81:10.11:3.67:72.10 (Figure 1B). The molar percentage of GalA exceeded 70%, which is consistent with the uronic acid content test results (79.80 ± 1.98%). The polysaccharides of *Schisandra* fruits of the same genus were previously reported to be mainly composed of GalA and xylose (11). These results suggested that KCFP is an acidic heteropolysaccharide composed of homogalacturonic acid.

#### Thermal and X-Ray Diffraction Analyses of *Kadsura coccinea* Fruit Polysaccharide

The thermogravimetry curves showed that KCFP underwent two major mass loss processes (Figure 1C). The first mass loss occurred at 60–190°C and was attributed to the loss of both free water and bound water absorbed in KCFP. The second mass loss occurred at 210–400°C, which was the mass loss caused by KCFP decomposition. When the temperature increased above 600 °C, the mass loss was gradual, and the mass of the remaining residue at 800 °C was 28.6%. Under a nitrogen atmosphere, this material is mainly carbon residue (12).

The derivative thermogravimetry curve (Figure 1C) showed two peaks in the range of 210–400°C. The peak with the highest intensity was observed at 232.3°C, indicating that the mass loss rate of KCFP was greatest at this temperature (4). In addition, the derivative thermogravimetry peak at 283.0°C may be due to the fact that KCFP consists of two main structural units. As the temperature increased, one of the polysaccharide units decomposed first at 231°C, while the other polysaccharide unit decomposed at 283°C (13). The thermal analysis results indicated that KCFP should not be subjected to temperatures greater than 210°C; otherwise, it will decompose.

X-Ray Diffraction is often used to determine the crystalline or amorphous properties of polymers. The pattern of KCFP had a broad diffraction peak at approximately 2θ = 22° and a shoulder peak at 2θ = 13° (Figure 1D), indicating an amorphous nature. The XRD pattern showed no sharp peaks, which indicated that KCFP does not contain crystalline impurities (14).

#### Observations of *Kadsura coccinea* Fruit Polysaccharide Morphology Using Scanning Electron Microscope and Atomic Force Microscopy

**FIGURE 2:**
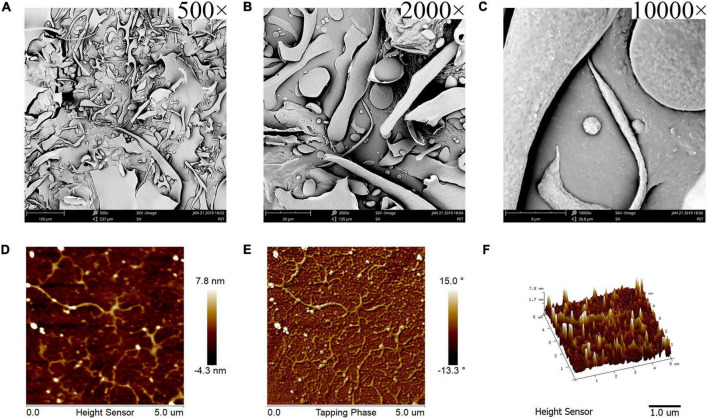
SEM and AFM images of KCFP **(A–C)** SEM images captured at 500×, 2000×, and 10,000× magnification, respectively. **(D–F)** AFM 2D, phase and 3D images, respectively. SEM, scanning electron microscopy; AFM, atomic force microscopy; KCFP, *Kadsura coccinea* fruit polysaccharide.

Scanning electron microscope micrographs of KCFP at different magnifications are shown in Figures 2A–C. At a magnification of 500×, KCFP generally displayed a sheet-like structure with some filament-like, rod-like and pebble-like features (Figure 2A). At a magnification of 2000×, the edges of the various shapes and structures were rounded (Figure 2B). When the magnification increased to 10000× (Figure 2C), dense and small convex features were clearly visible on the KCFP surface. KCFP has a rough surface and filamentous structure, which is probably related to the presence of various hydroxyl and carboxyl groups in the polysaccharide (15).

Atomic force microscopy is a powerful tool for observing the morphology of polysaccharides at the nanometre scale (16, 17). As shown in Figures 2D,E, KCFP was evenly distributed in the field of view, which allowed for easy observation of its morphology and structure. KCFP is composed of a main chain and multiple side chains, and these side chains exhibited clear irregular flexible extensions and a high degree of branching. The side chains are also cross-linked in places. These cross-links form a longer molecular chain, which increases the chain length and forms an irregular network structure that is similar to the simulated structure of pectin (18, 19). The 3D image in Figure 2F shows the roughness of KCFP, which is consistent with the results from the SEM observations.

#### Fourier Transform Infrared and Nuclear Magnetic Resonance Spectroscopy

**FIGURE 3:**
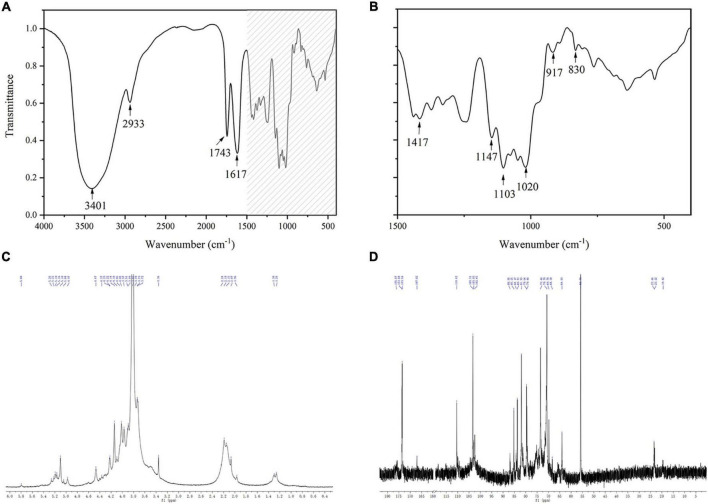
FTIR and 1D NMR spectra of KCFP **(A,B)** Infrared spectra. **(C,D)** ^1^H NMR and ^13^C NMR spectra, respectively. FTIR, Fourier transform infrared; NMR, nuclear magnetic resonance; KCFP, *Kadsura coccinea* fruit polysaccharide.

The structural characteristics of KCFP were further analyzed using FTIR and NMR spectroscopy (Figure 3). The strong peaks observed in the FTIR spectrum at 3401 cm^–1^ and 2934 cm^–1^ might be attributed to the stretching vibrations of O-H and C-H, respectively. In addition, the strong peak at 1020–1150 cm^–1^ was due to the stretching vibrations of the C-O-H and C-O-C moieties contained in rings (4). These peaks are the characteristic absorptions of carbohydrates, indicating that KCFP is a carbohydrate. The sharp peaks at 1743 cm^–1^ and 1651 cm^–1^ were assigned to the C = O stretching vibrations of methylesterified carboxyl groups and free carboxyl groups, respectively, which are characteristic peaks of pectic polysaccharides (20–22). Moreover, absorption bands at 1147, 1103, 1078, 1049, 1020, and 970 cm^–1^ were also observed in the previously reported pectin spectrum (23). Together, these findings suggest that KCFP is a pectin-like polysaccharide. In addition, the peak at 917 cm^–1^ suggested the presence of a glucopyranosyl moiety with a nonsymmetric ring (24), and the peaks at 830 cm^–1^ and 890 cm^–1^ suggested the presence of both α- and β-linkages (25, 26).

In the ^1^H NMR spectrum of KCFP, we observed signals for 8 anomeric protons at 5.80, 5.26/5.22, 5.19/5.16, 5.10, 5.04, 4.97, 4.60, and 4.47 ppm, implying the presence of 8 types of monosaccharide residues, which is consistent with our analysis of the monosaccharide composition. In addition, both α-type and β-type glycoside residues have been found in KCFP (27). The peaks at 3.82, 1.97–2.25 and 1.23/1.30 in the ^1^H NMR spectrum indicate the presence of methoxy groups, O-acetyl substituents and the C-6 methyl protons of rhamnose, respectively (28–30). Similar to the ^1^H NMR spectrum, certain structural characteristics of KCFP were also observed in the ^13^C NMR spectrum. For example, the signals with chemical shifts of 19.41 and 90–110 ppm were attributed to the methyl carbons of rhamnose and anomeric carbons, respectively (29, 31). The signal at 55.79 ppm was assigned to the O-methyl group, and the peak at 173.6 ppm was ascribed to the carboxy carbon (32). Moreover, some new structural characteristics were identified in KCFP from analysis of the ^13^C NMR spectrum. For example, the signal at ∼103.31 ppm was assigned to C-1 of GalA (29). In addition, the signal at 110.42 ppm was derived from C-1 of arabinose, indicating that arabinose is the main neutral sugar in KCFP (33). Taken together, these results show that KCFP is a typical pectin polysaccharide.

#### Rheological Properties of *Kadsura coccinea* Fruit Polysaccharide

**FIGURE 4:**
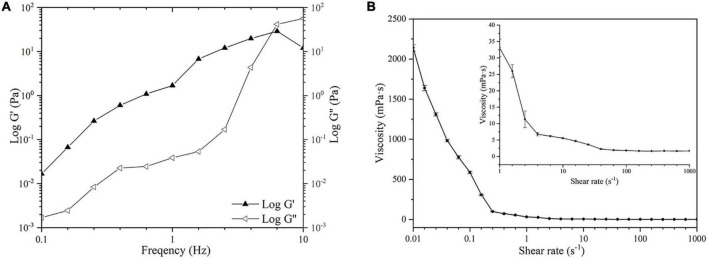
Rheological properties of KCFP **(A)** Frequency sweep **(B)** Flow curve. KCFP, *Kadsura coccinea* fruit polysaccharide.

The relationship between the storage modulus (G′) and loss modulus (G″) of a 5 mg/mL KCFP solution over the oscillation frequency range of 0.1 to 10 Hz is shown in Figure 4A. In the oscillation frequency range of 0.1–7.66 Hz, G′ > G,″ indicating that the solution exhibited weak gel properties. When the oscillation frequency was greater than 7.66 Hz (G′ < G″), the solution displayed fluid properties. The plots in Figure 4B show the relationship between the viscosity and the shear rate of the 5 mg/mL KCFP solution. At shear rates of 0.01–1000 s^–1^, the viscosity of the KCFP solution decreased with increasing shear rate, showing characteristics of shear thinning, which is typical of pseudoplastic fluids. When the shear rate was greater than 100 s^–1^, the characteristics of the solution approached those of a Newtonian fluid.

### Antioxidant and Hypolipidaemic Activity *in vitro*

#### 2,2-Diphenyl-1-Picrylhydrazyl Radical Scavenging Activity

**FIGURE 5:**
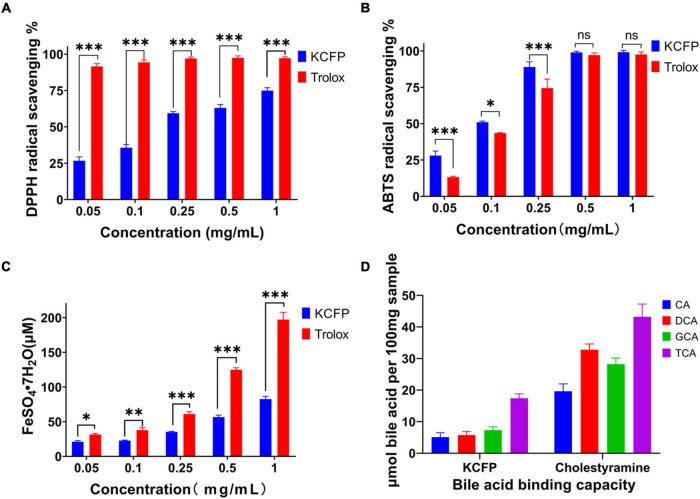
Antioxidant activity and bile acid binding capacity of KCFP (n=3; ns: not significant, *p < 0.05, **p < 0.01, and ***p < 0.001). **(A)** DPPH free radical scavenging ability. **(B)** ABTS free radical scavenging ability. **(C)** Total antioxidant capacity. **(D)** Bile acid binding capacity. DPPH, 2,2-Diphenyl-1-picrylhydrazyl; ABTS, 2,2′-azino-bis(3-ethylbenzothiazoline-6-sulfonic acid) diammonium salt; CA, cholic acid; DCA, deoxycholic acid; GCA, glycocholic acid; TCA, taurocholic acid; KCFP, *Kadsura coccinea* fruit polysaccharide.

Different concentrations of KCFP were used to scavenge DPPH free radicals, and the results are shown in Figure 5A. In this experiment, Trolox served as the positive control. At KCFP concentrations ranging from 0.05 to 1.0 mg/mL, the KCFP concentration and DPPH free radical scavenging activity were positively correlated; that is, as the KCFP concentration increased, the scavenging rate increased. Trolox at a concentration of 0.05 mg/mL had a scavenging rate of 91.6%, while the DPPH free radical scavenging rate of KCFP at this same concentration was very low at only 26.7%.

The concentration of KCFP required to obtain 50% of the maximum effect (IC_50_ value) determined from the DPPH free radical scavenging experiments was 0.150 mg/mL, which was calculated with GraphPad Prism 8.0 software (United States). This result is much lower than those of other pectin polysaccharides, such as the pectin extracted from jackfruit peel (IC_50_ = 1.1 mg/mL) (34), the pectic polysaccharide in apple pomace (IC_50_ = 4.69 mg/mL), commercial apple pectin (IC_50_ = 6.50 mg/mL) (35) and the pectin polysaccharide extracted from Guara fruits (IC_50_ = 10.8 mg/mL) (36). These data show that the DPPH free radical scavenging ability of KCFP is superior to those of pectin polysaccharides from other sources; thus, KCFP presents potential antioxidant application value.

### ABTS Free Radical Scavenging Ability

The ABTS radical scavenging abilities of KCFP and Trolox at concentrations ranging from 0.05 to 1.0 mg/mL are shown in Figure 5B. As shown in Figure 5B, the ABTS free radical scavenging abilities of both KCFP and Trolox increased with their increasing concentration. At concentrations ranging from 0.05 to 0.25 mg/mL, the ABTS free radical scavenging ability of KCFP was significantly greater than that of Trolox. When the sample concentration reached 0.5 mg/mL, the scavenging rates of both compounds were close to 100%. The IC_50_ value of KCFP in the ABTS free radical scavenging experimental system was 0.121 mg/mL, showing strong free radical scavenging ability.

#### Ferric Reducing Antioxidant Power

A FRAP assay was used to estimate the ability of the tested samples to withstand the transformation of Fe^3+^ to Fe^2+^ in the presence of tripyridyltriazine (TPTZ). At concentrations ranging from 10 to 500 μM, the concentration of FeSO_4_⋅7H_2_O showed a linear relationship with the absorbance of the sample. This linear equation was C = 420.0A–13.101, and the correlation coefficient (R^2^) was 0.9989. The antioxidant capacities of the KCFP samples were calculated from this equation and are reported as the concentration of FeSO_4_⋅7H_2_O standard solution (μM FeSO_4_⋅7H_2_O) (Figure 5C). The FRAP experiment showed that the total antioxidant capacities of different concentrations of KCFP and Trolox were different and increased with increasing concentration. Notably, when KCFP and Trolox were analyzed at the same concentration, the antioxidant capacity of KCFP was inferior to that of the positive control Trolox. This trend is similar to the trend reported by Liu et al. (10).

#### Bile Acid Binding Capacity

Cholic acid, DCA, GCA and TCA are the main bile acids synthesized in the human body. Many studies have shown that polysaccharides and soluble dietary fiber lower blood lipid levels, especially serum TC and LDL-C levels (37). Figure 5D shows the bile acid binding capacity of KCFP with cholestyramine (CT) serving as a positive control. On an equal DM basis, the bile acid binding capacity of CT was significantly higher than that of KCFP. Under the same experimental conditions, the abilities of KCFP to bind CA, DCA, GCA and TCA were 32.78, 20.59, 41.31, and 44.15% those of CT, respectively. CT, a lipid-lowering drug, interacts with bile acids to facilitate intestinal excretion, which in turn stimulates the conversion of cholesterol into bile acids in the liver, reducing TC and LDL-C levels. The mechanism by which polysaccharides lower blood lipid levels may be similar to that of CT (38).

The biological activity of polysaccharides is closely related to their structural characteristics, including monosaccharide composition, molecular weight, glycosidic linkages and polysaccharide conformation (10). Uronic acid content is generally considered an important indicator of the antioxidant capacity of polysaccharides; namely, polysaccharides containing high contents of uronic acid usually exhibit robust antioxidant activities (10, 39, 40). The mechanism by which pectin polysaccharides scavenge free radicals is that the hydrogen atom of the hydroxyl group of GalA reacts with the electron of the free radical through electron transfer. A stronger interaction between the hydrogen atoms and electrons results in increased antioxidant activity of the pectin polysaccharides (41). Therefore, the great antioxidant activity of KCFP has a close relationship with its high GalA content (79.8%). A bile acid binding experiment was conducted to evaluate the potential hypolipidaemic activity of KCFP. The bile acid binding capacity of polysaccharides is related to their molecular weight, characteristic groups, rheological properties, solution conformation and other factors (4, 38). The results of this study showed that KCFP has the ability to bind bile acids. Although KCFP could not bind bile acids as well as the positive control (CT resin), KCFP still shows certain potential hypolipidaemic effects.

Hyperlipidaemia is one of the main risks of cardiovascular disease (42). Research has shown that polysaccharides generally exert both antioxidant and hypolipidaemic effects (43, 44). Recent studies have suggested that hyperlipidaemia occurs due to oxidative stress and that the hypolipidaemic activity of polysaccharides is attributable to their antioxidant capacity (45). KCFP was shown here to have better antioxidant activity than most polysaccharides in addition to its good ability to bind bile acids *in vitro*. Therefore, the *in vivo* antioxidant and hypolipidaemic effects of KCFP are worthy of further study.

### Antioxidant and Hypolipidaemic Activities of *Kadsura coccinea* Fruit Polysaccharide in High-Fat Diet-Induced Hyperlipidaemic Mice

A high-fat diet causes oxidative damage in the body (46), increases serum and liver levels of TC, TG, and LDL-C, and reduces HDL-C contents, which may lead to cardiovascular disease (47, 48). Thus, the antioxidant and hypolipidaemic activities of KCFP were investigated by analyzing liver tissue sections, liver antioxidant enzymes, and liver and serum lipid levels.

**FIGURE 6:**
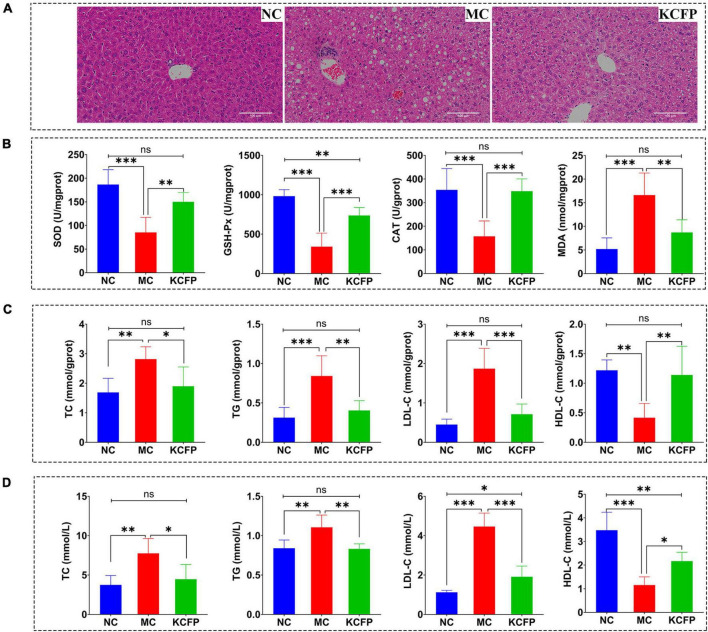
Effects of KCFP on high-fat diet-induced hyperlipidaemia in C57BL/6N mice (*n* = 6; ns: not significant, *: *p* < 0.05, ^**^: *p* < 0.01, and ^***^: *p* < 0.001). **(A)** H&E-stained sections of liver tissue (200×). **(B)** Hepatic antioxidant enzyme activity. **(C)** Liver lipid levels. **(D)** Serum lipid levels. NC, normal control; MC, model control; KCFP, *Kadsura coccinea* fruit polysaccharide; TC, total cholesterol; TG, triglyceride; LDL-C, low-density lipoprotein cholesterol; HDL-C, high-density lipoprotein cholesterol; GSH-Px, glutathione peroxidase; SOD, superoxide dismutase; CAT, catalase; MDA, malondialdehyde.

Histopathological examination of the mouse liver tissue showed that KCFP intervention significantly reduced the symptoms of liver injury, fat accumulation and inflammation induced by a high-fat diet, as shown in Figure 6A. The effect of KCFP on the hepatic antioxidant enzyme activity in high-fat diet-fed mice is shown in Figure 6B. Compared with the NC group, the activities of SOD, CAT and GSH-Px were significantly decreased in the liver tissue from the mice in the MC group (*p* < 0.001). After KCFP intervention, the activities of SOD, CAT and GSH-Px were significantly increased compared with those in the MC group (*p* < 0.01). In addition, the MDA level in the MC group was significantly higher than that in the NC group (*p* < 0.001). Although KCFP intervention decreased the MDA concentration, a significant difference was not observed between the KCFP and NC groups (*p* > 0.05).

The effects of KCFP on liver and serum lipid levels are shown in Figures 6C,D. In this study, the levels of TC, TGs and LDL-C in the mice in the MC group were significantly higher than those in the NC group (*p* < 0.01), while the HDL-C level was significantly lower than that in the NC group (*p* < 0.01). After 8 weeks of KCFP intervention, the serum and liver levels of TC, TG and LDL-C in the mice in the KCFP group decreased significantly compared with those in the MC group (*p* < 0.05), and the level of HDL-C increased significantly (*p* < 0.05). The serum TC and TG levels in the mice in the KCFP group were restored to those in the NC group; similarly, the TC, TG, LDL-C and HDL-C levels in the livers of the KCFP group of mice also returned to those of the NC group (*p* > 0.05). These results fully indicate that KCFP possesses significant hypolipidaemic activity *in vivo*, which is consistent with previous reports on the hypolipidaemic activity of pectin polysaccharides (41, 44, 49).

Previous studies have shown that polysaccharides can correct the disorder of free radical metabolism, maintain the dynamic balance of oxidative and antioxidant systems, reduce the toxic side effects of free radicals, and decrease the damage to the body caused by lipid peroxidation (50). Therefore, the hypolipidaemic effects of KCFP may be achieved by regulating antioxidant enzyme activity.

## Conclusion

Our results suggested that KCFP exhibited the characteristics of a pectin-like polysaccharide. In addition, KCFP showed strong antioxidant and hypolipidaemic activities both *in vitro* and *in vivo*, which may be related to its high uronic acid content and highly branched structure. Our study extends the understanding of the physicochemical characteristics and antioxidant and hypolipidaemic activities of KCFP, but its exact structure and mechanism of action should be investigated in further studies.

## Data Availability Statement

The original contributions presented in the study are included in the article/supplementary material, further inquiries can be directed to the corresponding authors.

## Ethics Statement

The animal study was reviewed and approved by Committee on the Ethics of Animal Experiments of Guangxi Botanical Garden of Medicinal Plants.

## Author Contributions

HL and XX: conceptualization. HL: methodology, writing—original draft preparation, and visualization. QC and SW: software. XX, SL, and LW: validation. ZZ: formal analysis. HL, XX, SL, and LW: investigation. XX: resources and supervision. XG: data curation. XG and ZZ: writing—review and editing. SL: project administration. HL, TW, and ZZ: funding acquisition. All authors have read and agreed to the published version of the manuscript.

## Conflict of Interest

The authors declare that the research was conducted in the absence of any commercial or financial relationships that could be construed as a potential conflict of interest.

## Publisher’s Note

All claims expressed in this article are solely those of the authors and do not necessarily represent those of their affiliated organizations, or those of the publisher, the editors and the reviewers. Any product that may be evaluated in this article, or claim that may be made by its manufacturer, is not guaranteed or endorsed by the publisher.

## References

[1] Dong Y Qi Y Liu M Song X Zhang C Jiao X Antioxidant, anti-hyperlipidemia and hepatic protection of enzyme-assisted *Morehella esculenta* polysaccharide. *Int J Biol Macromol.* (2018) 120:1490–9. 10.1016/j.ijbiomac.2018.09.134 30266646

[2] Bian Y Li X Li X Ju J Liang H Hu X Daming capsule, a hypolipidaemic drug, lowers blood lipids by activating the ampk signalling pathway. *Biomed Pharmacother.* (2019) 117:109176. 10.1016/j.biopha.2019.109176 31387185

[3] Okopień B Bułdak A Bołdys A. Benefits and risks of the treatment with fibrates—-a comprehensive summary. *Expert Rev Clin Pharmacol.* (2018) 11:1099–112. 10.1080/17512433.2018.1537780 30328735

[4] Long H Gu X Zhou N Zhu Z Wang C Liu X Physicochemical characterization and bile acid-binding capacity of water-extract polysaccharides fractionated by stepwise ethanol precipitation from *Caulerpa lentillifera*. *Int J Biol Macromol.* (2020) 150:654–61. 10.1016/j.ijbiomac.2020.02.121 32061693

[5] Wang L Liao S Long H Xia X Chen Q Liang J Analysis and evaluation of nutritional components of *Kadsura coccinea* fruit peel and pulp (in chinese). *Food Ferment Industr.* (2021) 47:124–31. 10.13995/j.cnki.11-1802/ts.025882

[6] Long H Gu X Zhu Z Wang C Xia X Zhou N Effects of bottom sediment on the accumulation of nutrients in the edible green seaweed *Caulerpa lentillifera* (sea grapes). *J Appl Phycol.* (2020) 32:705–16. 10.1007/s10811-019-01949-9

[7] Chen Y Xie M Nie S Li C Wang Y. Purification, composition analysis and antioxidant activity of a polysaccharide from the fruiting bodies of *Ganoderma atrum*. *Food Chem.* (2008) 107:231–41. 10.1016/j.foodchem.2007.08.021

[8] Li L Thakur K Liao B Zhang J Wei Z. Antioxidant and antimicrobial potential of polysaccharides sequentially extracted from *Polygonatum cyrtonema* Hua. *Int J Biol Macromol.* (2018) 114:317–23. 10.1016/j.ijbiomac.2018.03.121 29578016

[9] Benzie IFF Strain JJ. The ferric reducing ability of plasma (FRAP) as a measure of “antioxidant power”: the FRAP assay. *Anal Biochem.* (1996) 239:70–6. 10.1006/abio.1996.0292 8660627

[10] Liu X Liu H Yan Y Fan L Yang J Wang X Structural characterization and antioxidant activity of polysaccharides extracted from jujube using subcritical water. *LWT.* (2020) 117:108645. 10.1016/j.lwt.2019.108645

[11] Wang H Jiang H Wang S Li X Yao D Dong J Extraction, purification and preliminary characterization of polysaccharides from *Kadsura marmorata* fruits. *Carbohydr Polym.* (2013) 92:1901–7. 10.1016/j.carbpol.2012.11.060 23399235

[12] Vazquez A Foresti ML Cerrutti P Galvagno M. Bacterial cellulose from simple and low cost production media by *Gluconacetobacter xylinus*. *J Polym Environ.* (2013) 21:545–54. 10.1007/s10924-012-0541-3

[13] Yang H Wang D Deng J Yang J Shi C Zhou F Activity and structural characteristics of peach gum exudates. *Int J Polym Sci.* (2018) 2018:1–5. 10.1155/2018/4593735

[14] Kouadri I Layachi A Makhlouf A Satha H. Optimization of extraction process and characterization of water-soluble polysaccharide (Galactomannan) from algerian biomass; *Citrullus colocynthis* seeds. *Int J Polym Anal Chem.* (2018) 23:362–75. 10.1080/1023666X.2018.1455343

[15] Ji X Cheng Y Tian J Zhang S Jing Y Shi M. Structural characterization of polysaccharide from jujube (*Ziziphus jujuba* Mill.) Fruit. *Chem Biol Technol Agric.* (2021) 8:54. 10.1186/s40538-021-00255-2

[16] Liu W Wang H Yu J Liu Y Lu W Chai Y Structure, chain conformation, and immunomodulatory activity of the polysaccharide purified from *Bacillus* Calmette Guerin formulation. *Carbohyd Polym.* (2016) 150:149–58. 10.1016/j.carbpol.2016.05.011 27312624

[17] Wang J Nie S. Application of atomic force microscopy in microscopic analysis of polysaccharide. *Trends Food Sci Tech.* (2019) 87:35–46. 10.1016/j.tifs.2018.02.005

[18] Wu HC Bulgakov VP Jinn TL. Pectin methylesterases: cell wall remodeling proteins are required for plant response to heat stress. *Front Plant Sci.* (2018) 9:1612. 10.3389/fpls.2018.01612 30459794 PMC6232315

[19] Cohen E Merzendorfer H. *Extracellular sugar-based biopolymers matrices.* (Vol. 12). Cham: Springer (2019). p. 487.

[20] Jiang Y Xu Y Li F Li D Huang Q. Pectin extracted from persimmon peel: a physicochemical characterization and emulsifying properties evaluation. *Food Hydrocoll.* (2020) 101:105561. 10.1016/j.foodhyd.2019.105561

[21] Yang X Nisar T Hou Y Gou X Sun L Guo Y. Pomegranate peel pectin can be used as an effective emulsifier. *Food Hydrocoll.* (2018) 85:30–8. 10.1016/j.foodhyd.2018.06.042

[22] Matharu AS Houghton JA Lucas-Torres C Moreno A. Acid-free microwave-assisted hydrothermal extraction of pectin and porous cellulose from mango peel waste – towards a zero waste mango biorefinery. *Green Chem.* (2016) 18:5280–7. 10.1039/C6GC01178K

[23] Kačuráková M Capek P Sasinková V Wellner N Ebringerová A. FT-IR study of plant cell wall model compounds: pectic polysaccharides and hemicelluloses. *Carbohyd Polym* (2000) 43:195–203. 10.1016/S0144-8617(00)00151-X

[24] Wang P Huang Q Chen C You L Liu RH Luo Z The chemical structure and biological activities of a novel polysaccharide obtained from *Fructus mori* and its zinc derivative. *J Funct Foods.* (2019) 54:64–73. 10.1016/j.jff.2019.01.008

[25] Wang N Zhang Y Wang X Huang X Fei Y Yu Y Antioxidant property of water-soluble polysaccharides from poria cocos Wolf using different extraction methods. *Int J Biol Macromol.* (2016) 83:103–10. 10.1016/j.ijbiomac.2015.11.032 26601761

[26] Chylińska M Szymańska-Chargot M Zdunek A. FT-IR and FT-Raman characterization of non-cellulosic polysaccharides fractions isolated from plant cell wall. *Carbohyd Polym.* (2016) 154:48–54. 10.1016/j.carbpol.2016.07.121 27577895

[27] Su Y Li L. Structural characterization and antioxidant activity of polysaccharide from four auriculariales. *Carbohyd Polym.* (2020) 229:115407. 10.1016/j.carbpol.2019.115407 31826485

[28] Gao X Qu H Shan S Song C Baranenko D Li Y A novel polysaccharide isolated from *Ulva pertusa*: structure and physicochemical property. *Carbohyd Polym.* (2020) 233:115849. 10.1016/j.carbpol.2020.115849 32059900

[29] Shakhmatov EG Toukach PV Makarova EN. Structural studies of the pectic polysaccharide from fruits of *Punica granatum*. *Carbohyd Polym.* (2020) 235:115978. 10.1016/j.carbpol.2020.115978 32122509

[30] Guo Q Du J Jiang Y Goff HD Cui SW. Pectic polysaccharides from hawthorn: physicochemical and partial structural characterization. *Food Hydrocoll.* (2019) 90:146–53. 10.1016/j.foodhyd.2018.10.011

[31] Zhao J Zhang F Liu X St. Ange K Zhang A Li Q Isolation of a lectin binding rhamnogalacturonan-I containing pectic polysaccharide from pumpkin. *Carbohyd Polym.* (2017) 163:330–6. 10.1016/j.carbpol.2017.01.067 28267513

[32] Cheng HN. NMR analysis of compositional heterogeneity in polysaccharides. *Pure Appl Chem.* (2017) 89:877–83. 10.1515/pac-2016-1020

[33] Zou S Zhang X Yao W Niu Y Gao X. Structure characterization and hypoglycemic activity of a polysaccharide isolated from the fruit of *Lycium barbarum* L. *Carbohyd Polym.* (2010) 80:1161–7. 10.1016/j.carbpol.2010.01.038

[34] Xu S Liu J Huang X Du L Shi F Dong R Ultrasonic-microwave assisted extraction, characterization and biological activity of pectin from jackfruit peel. *LWT.* (2018) 90:577–82. 10.1016/j.lwt.2018.01.007

[35] Wang X Lü X. Characterization of pectic polysaccharides extracted from apple pomace by hot-compressed water. *Carbohyd Polym.* (2014) 102:174–84. 10.1016/j.carbpol.2013.11.012 24507270

[36] Hua D Zhang D Huang B Yi P Yan C. Structural characterization and DPPH radical scavenging activity of a polysaccharide from Guara fruits. *Carbohyd Polym.* (2014) 103:143–7. 10.1016/j.carbpol.2013.12.009 24528712

[37] Gunness P Gidley MJ. Mechanisms underlying the cholesterol-lowering properties of soluble dietary fibre polysaccharides. *Food Funct.* (2010) 1:149. 10.1039/c0fo00080a 21776465

[38] Gao J Lin L Sun B Zhao M. A comparison study on polysaccharides extracted from *Laminaria japonica* using different methods: structural characterization and bile acid-binding capacity. *Food Funct.* (2017) 8:3043–52. 10.1039/C7FO00218A 28805835

[39] Zhu R Zhang X Wang Y Zhang L Zhao J Chen G Characterization of polysaccharide fractions from fruit of *Actinidia arguta* and assessment of their antioxidant and antiglycated activities. *Carbohyd Polym.* (2019) 210:73–84. 10.1016/j.carbpol.2019.01.037 30732783

[40] Wang CC Chang SC Inbaraj BS Chen BH. Isolation of carotenoids, flavonoids and polysaccharides from Lycium barbarum L. and evaluation of antioxidant activity. *Food Chem.* (2010) 120:184–92. 10.1016/j.foodchem.2009.10.005

[41] Zeng H Miao S Zhang Y Lin S Jian Y Tian Y Isolation, preliminary structural characterization and hypolipidemic effect of polysaccharide fractions from *Fortunella margarita* (Lour.) Swingle. *Food Hydrocoll.* (2016) 52:126–36. 10.1016/j.foodhyd.2015.05.028

[42] Nelson RH. Hyperlipidemia as a risk factor for cardiovascular disease. *Prim Care Clin Off Pract.* (2013) 40:195–211. 10.1016/j.pop.2012.11.003 23402469 PMC3572442

[43] Xu Y Zhang X Yan X Zhang J Wang L Xue H Characterization, hypolipidemic and antioxidant activities of degraded polysaccharides from *Ganoderma lucidum*. *Int J Biol Macromol.* (2019) 135:706–16. 10.1016/j.ijbiomac.2019.05.166 31129213

[44] Rjeibi I Feriani A Hentati F Hfaiedh N Michaud P Pierre G. Structural characterization of water-soluble polysaccharides from *Nitraria retusa* fruits and their antioxidant and hypolipidemic activities. *Int J Biol Macromol.* (2019) 129:422–32. 10.1016/j.ijbiomac.2019.02.049 30742925

[45] Tang Z Gao H Wang S Wen S Qin S. Hypolipidemic and antioxidant properties of a polysaccharide fraction from *Enteromorpha prolifera*. *Int J Biol Macromol.* (2013) 58:186–9. 10.1016/j.ijbiomac.2013.03.048 23541551

[46] Zhu Z Lin Z Jiang H Jiang Y Zhao M Liu X. Hypolipidemic effect of Youcha in hyperlipidemia rats induced by high-fat diet. *Food Funct.* (2017) 8:1680–7. 10.1039/C7FO00089H 28379241

[47] Zhang Y Wang Z Jin G Yang X Zhou H. Regulating dyslipidemia effect of polysaccharides from *Pleurotus ostreatus* on fat-emulsion-induced hyperlipidemia rats. *Int J Biol Macromol.* (2017) 101:107–16. 10.1016/j.ijbiomac.2017.03.084 28322967

[48] Zhu Z Zhu B Sun Y Ai C Wang L Wen C Sulfated polysaccharide from sea cucumber and its depolymerized derivative prevent obesity in association with modification of gut microbiota in high-fat diet-fed mice. *Mol Nutr Food Res.* (2018) 62:1800446. 10.1002/mnfr.201800446 30267558

[49] Ru Y Chen X Wang J Guo L Lin Z Peng X Polysaccharides from *Tetrastigma hemsleyanum* Diels et Gilg: extraction optimization, structural characterizations, antioxidant and antihyperlipidemic activities in hyperlipidemic mice. *Int J Biol Macromol.* (2019) 125:1033–41. 10.1016/j.ijbiomac.2018.11.236 30500505

[50] Feng L Yu C Ying K Hua J Dai X. Hypolipidemic and antioxidant effects of total flavonoids of *Perilla frutescens* leaves in hyperlipidemia rats induced by high-fat diet. *Food Res Int.* (2011) 44:404–9. 10.1016/j.foodres.2010.09.035

